# Difficulties of unit managers in selected district hospitals in Cameroon

**DOI:** 10.4102/curationis.v42i1.1993

**Published:** 2019-10-10

**Authors:** Esther L. Wanko Keutchafo, Jane Kerr

**Affiliations:** 1 School of Nursing and Public Health, University of KwaZulu-Natal, Durban, South Africa

**Keywords:** Cameroon, difficulties, district hospitals, unit managers, phenomenology

## Abstract

**Background:**

Being appointed to a managerial position because of one’s clinical skills seems to be prestigious, even powerful. However, being a unit manager in a resource-constrained district hospital can be a daunting task. Also, managing a ward unit with no previous training in leadership and management can be very challenging.

**Objectives:**

The purpose of this study was to describe the difficulties, in the day-to-day activities, of unit managers in selected Cameroonian district hospitals.

**Method:**

A constructionist, descriptive Husserlian phenomenological inquiry was conducted to describe the difficulties of unit managers in two district hospitals. Ten unit managers were selected through a purposive sampling scheme, and then interviewed using semi-structured interviews. Coliazzi’s qualitative data analysis method was used for analysis.

**Results:**

This study revealed that unit managers looked for assistance because it is not easy to be in their position. Their role implied facing difficulties and making sacrifices for something that is not even worth the trouble. Therefore, as a way to overcome their difficulties, they asked for assistance from the organisation, from their families and from God as strategies to face their difficulties.

**Conclusion:**

The difficulties faced by unit managers in the selected district hospitals revealed the need to prepare nurses for managerial positions by ensuring they are trained as managers before commencing employment as a manager.

## Background

Cameroon is a lower middle-income country at the heart of the Gulf of Guinea in Central Africa (Mboti 2009). The healthcare system is pyramidal, comprising three levels, consisting of 4351 healthcare facilities in 2010 (Direction Des Resources Humaines Minsanté 2011). It aims to improve the health of the population (Ngwakongnwi, Suh & Quan 2014) by upscaling accessibility to quality and integrated care with the full participation of communities in the management and financing of health activities (Ngah et al. 2013). Yet, the system has some critical difficulties that include a poor health information system, is highly centralised and is underutilised at a peripheral level. Added to which, there are structural and capacity-building problems in health service administration at peripheral and central levels, and inadequacy of training of the health workforce (Nzima 2014), including nurses.

Nurses constitute the majority of the global healthcare workers (Campbell et al. 2013; Cross 2013) and unit managers play a pivotal role in any healthcare setting (Cziraki et al. 2014). However, there is a global nursing shortage (Ugochukwu et al. 2013) that includes the shortage of unit managers (Cross 2013). In Cameroon, unit managers in a district hospital are required to ensure the effective functioning of the unit (cleanliness, daily usage of the premises, the stocks, and the supplies). They are also expected to manage the staff nurses (uniform, punctuality, accuity continuous education, and short leave). They monitor the nursing care and ensure the delivery of quality care. They are involved in administration, nursing student supervision, management of conflict between patients, patients’ families and staff; apply the resolutions and instructions given during monthly meetings and daily meetings with staff; and notify management of any unusual situations that may occur in the unit. These constitute the many role demands experienced by the unit managers in the context of the study.

## Problem statement

The role of unit managers has evolved from a head nurse position with responsibility for nursing staff and practice to a management position that interfaces with educators, clinical nurse specialists, physicians and non-nursing personnel (Cziraki et al. 2014). Unit managers play a pivotal role in changing the environment of acute care hospitals (Cadmus & Wisniewska 2013). They have many role demands (Brown et al. 2013) and play multidimensional roles (Miri et al. 2014) such as the coordination of care activities (Kerr, Brysiewicz & Bhengu 2014) and the running of the unit (Matlakala, Bezuidenhout & Botha 2014). Their responsibilities also include looking after the interest of the staff nurses (Kerr et al. 2014) and ensuring optimal patient outcomes and satisfaction (Cadmus & Wisniewska 2013; Wong & Laschinger 2015).

Some unit managers perceive themselves as having many tasks and duties for which they feel ill-prepared (Udod & Care 2013). In fact, some unit managers find themselves in the situation of being promoted from the bedside role to a role in which they are in charge of clinical, financial and significantly increased personnel management duties (Townsend, Wilkinson & Kellner 2015). Their many role demands make their role difficult and challenging. The situation is worsened when the unit managers did not receive any training in nursing management prior to appointment. Given the resource-constrained working environment in Cameroon, there is a need to obtain insight into the unit managers’ difficulties. The rationale is that the quality of care and organisational goals can be compromised if unit managers develop their leadership skills through work experience only and not by formal management training. In addition, unit managers could feel overwhelmed if they do not have the relevant skills to manage the unit.

## Aim and objectives

The aim of the study was to describe unit managers’ lived experiences in selected district hospitals in Cameroon. The objectives were to describe the day-to-day practice of unit managers, to describe the difficulties faced during their day-to-day practices and to identify suggestions for the improvement of unit managers’ efficiency in Cameroon. This article reports on the difficulties faced during day-to-day practice.

## Definition of key concepts

The following concepts are considered in this article:

- Unit manager refers to registered nurses in a position of line responsibility for nursing, with staff nurses reporting directly to them (Laschinger et al. 2008). They are a consistent presence, uniquely positioned within the frontline of the intricacies of nurse–patient, nurse–medical practitioner and nurse–interdisciplinary team dynamics (Baker et al. 2012). They can be called frontline nurse managers, first-line nurse managers, unit managers, ward managers or ward charges.
- District hospitals refer to healthcare facilities that are situated at the peripheral level of the three-level Cameroonian healthcare system. They are assigned the role of interface between the community and health services, and are supposed to serve as the locus for integrated, continuous and comprehensive healthcare (Zamo-Akono, Ndjokou & Song-Ntamack 2013).

## Research methods and design

### Study design

A qualitative descriptive design, using Husserlian phenomenology (Giorgi 2008), was conducted in selected hospitals in Cameroon. The design was chosen to discover what it is like to be a unit manager in a district hospital in Cameroon, excluding any mention of social, cultural or political contexts of the participants.

### Setting

The study focused on two district hospitals in Yaoundé, Cameroon. Yaoundé was selected because it is the best-served city in terms of health workers with 3.2 health workers per 1000 population (Ngah et al. 2013).

### Study population and sampling strategy

Unit managers working in district hospitals formed the target population. Unit managers were selected because they play a key role in coordinating patient care activities and in ensuring safety and quality care in hospital wards (Armstrong, Rispel & Penn-Kekana 2015). A convenience sampling scheme was used to select 10 unit managers who met the inclusion criteria. The sample size was decided upon reaching saturation when no new theme emerged from the data and when repetition occurred. The unit managers included those with a diploma or a degree in nursing, who had any experience in a managerial position, and who voluntarily agreed to participate in the study.

### Data collection

Data were collected through semi-structured interviews with open-ended questions to ensure a broad coverage of the issue. Yet, the interviews were not guided by the researcher, and focusing, not leading, questions were asked for clarification or elaboration.

### Data analysis

All the interviews were transcribed verbatim. The eight interviews conducted in French were transcribed and translated into English using the back-to-back translation method by a certified translator to allow peer review. The seven steps of Coliazzi’s method were used to analyse the data. In accordance with the method, the transcripts were read and re-read, and significant statements were extracted. Then meanings were formulated, categorised into theme clusters and finally validated with the original texts. The results were integrated into an exhaustive phenomena description, then reduced to the essential phenomenon structure and returned to the participants for validation.

### Trustworthiness

Trustworthiness was ensured by paying attention to four criteria. Credibility of the study was achieved through individual interviews with unit managers and the sharing of the transcripts with them. Dependability was ensured by the use of peer debriefing and member checks, as suggested by Shenton (2004). Transferability was ensured by the provision of extensive information for the application of this study to different settings. Details about the settings, any restrictions affecting the participants and the number of those involved, the number and length of the data collection sessions, and the time over which the data were collected were provided. Conformability was ensured by audio-recording and verbatim presentation of the data.

### Ethical considerations

Ethical clearance was obtained from the Humanities and Social Sciences Ethics Committee of the University of KwaZulu-Natal, clearance number HSS/1047/015M.

## Results

Ten unit managers agreed to participate in the study. Table 1 displays the socio-demographic characteristics of the participants. Overall, the participants of this study admitted that being a unit manager implied many responsibilities. They described their role as being ‘a mother of a family’ and viewed the staff nurses as their children. In relation to their difficulties, the theme that emerged from the data was ‘it is not easy’.

**TABLE 1:** Socio-demographic characteristics of the participants.

Code	Gender	Age group	Unit	Nursing degree	Years of working experience	Months in the position	Previous experience in nursing management	Trained as nurse manager
P1	Female	55–65	Surgical	DN	35	36	Yes	No
P2	Female	55–65	Maternity	MSN	23	48	Yes	No
P3	Female	25–35	Paediatric	DN	13	36	Yes	No
P4	Male	25–35	Gynaecology	DN	11	4	No	No
P5	Female	25–35	Medical	MSc	5	5	No	No
P6	Female	35–45	Medical	DN	9	5	Yes	No
P7	Female	35–45	Maternity	DN	19	12	Yes	No
P8	Male	35–45	Paediatric	DN	9	4	No	No
P9	Female	25–35	Surgical	BSN	6	12	No	No
P10	Female	25–35	Medical	BSN	5	5	Yes	No

DN, Diploma Nurses; MSN, Master of Nursing; BSN, Bachelor of Nursing.

### ‘It is not easy’

This theme comprised three sub-themes: ‘facing difficulties’, ‘making sacrifices’, and ‘it is not worth it’, as summarised in Table 2.

**TABLE 2:** Themes and sub-themes.

Themes	Sub-themes	Categories
‘It is not easy’	‘Facing difficulties’	Patient-related difficulties Financially limited patients
		Staff nurse-related difficulties Difficult to lead their staff Could not correct the staff properly Working conditions of the staff Illegal sale of drugs
		Organisation-related difficulties Lack of space Lack of training in leadership and management
	‘Making sacrifices’	Dropping other income generating activities
		Going extra miles
		Buying material and supplies with their own money
	‘It is not worth it’	Insufficient financial motivation
Assistance from others	Assistance form the organisation	Support from the head nurse
	Assistance from their family	Help from their family members
	Assistance from God	Prayers

#### ‘Facing difficulties’

The participants expressed that there were difficulties faced on a daily basis as unit managers. For some participants, the difficulties they faced were patient related. One unit manager claimed that patients hindered them from working properly because of the patients’ financial limitations, as can be seen in the quotation below:

‘The first difficulty is having financially limited patients. The patient who comes to the district hospital is limited financially; he prevents you from treating him because he is unable to buy drugs or have a test.’ (P5, female, 5 months in the position)

In Cameroon, there is no medical aid scheme and every patient has to pay for everything from the consultation to the discharge in public hospitals. In other words, if patients do not pay for a syringe, they will not receive an injection. That is the reason why the unit managers had difficulty attending to patients with limited finances.

For other unit managers, their difficulties were related to the staff nurse. They expressed having trouble leading staff:

‘It is not easy to lead people; it is the first difficulty. You say as many times as possible: do not do it like this, do it like that; as soon as you turn your back, they juggle; they turn things around.’ (P3, female, 36 months in the position)

The participants cited reasons why it was difficult to lead their staff. Some mentioned that the nurses did not listen and comply with the instructions as illustrated by the following quote:

‘There is also a problem of consciousness: all the time, we have to shout at those who come late, who leave without any authorisation, leaving two colleagues in the unit. There are also misunderstandings because some do not always understand when I give instructions. People do not easily adhere to instructions; I struggle a lot to make people understand how I would like them to do their jobs.’ (P7, female, 12 months in the position)

Other participants said they could not correct the staff properly because they are dealing with adults:

‘Here we are dealing with adults; we are not dealing with children, thus, when something is wrong, you cannot take a cane to whip them. We have to talk as grown-up people … I am the youngest here. I deal with my elders and mothers. Do you think that I can scold them? If they do something wrong, even if it causes the death of a patient, I cannot shout at them because they are adults… You don’t reproach adults anyhow my daughter; moreover, when you are the younger one, they will give you poison.’ (P5, female, 5 months in the position)

‘It is not always easy, people go into a sulk every time; you tell them to do something, they don’t; but since they are adults, I have to repeat every time… Though it is pasted everywhere that the illegal sale of drugs is forbidden; but they always have drugs in their bags. I know about it, but I cannot hit anyone.’ (P8, male, 4 months in the position)

For another participant, it is difficult to lead people because a ward charge should not be strict when one considers the working conditions of the staff:

‘For someone who is not authoritarian enough, it is tough; and others seem to believe that you are soft. Whereas at the same time, you should be flexible because you should consider the conditions in which people are working. It is not always the ideal, we struggle. The payment also is not good; that is why even when you want to be so strict, at a certain moment you let go because it is not always easy.’ (P3, female, 36 months in the position)

Another issue related to the staff nurses was the illegal sale of drugs.

‘Though it is pasted everywhere that the illegal sale of drugs is forbidden; but they always have drugs in their bags. I know about it.… The Director himself is aware of this situation.… For example, a nurse who was on duty during the day may have sold drugs for a 24-hour treatment to a patient; the one on duty in the evening comes and refuses to treat the patient saying that: why did the other nurse sell drugs for the whole day’s treatment to the patient?’ (P8, male, 4 months in the position)

The participants also complained about the organisation. They mentioned the lack of material and supplies. The healthcare workers cannot perform procedures when there are insufficient resources.

‘In order to improve on my job, we need material and supplies because without them it is difficult.’ (P8, male, 4 months in the position)

‘We do not even have enough materials to work with, which is why you may notice that sometimes, when we are performing operations, we wash things, we sterilise them afterwards.’ (P10, female, 5 months in the position)

‘I work without gloves; there is no soap; the bathroom is not close; there is no tap to wash hands.’ (P8, male, 4 months in the position)

They also mentioned the lack of space in the premises. When the premises are small, the unit managers cannot ensure confidentiality anymore.

‘We do not have an office, I am not the only one, and none of the ward charges has an office. They will tell you that there is no space. We work in the presence of patients and staff. When you have to take a decision, it is difficult because it is with everybody present. There are moments where you need to have some privacy to take certain decisions; but the setting is small.’ (P3, female, 36 months in the position)

The lack of training in leadership and management was a further difficulty. One participant said:

‘I think that there could be small continuous training to guide people in their task, to tell them that as ward charges this is what is expected from you.’ (P3, female, 36 months in the position)

‘If there were appropriate training, it would be good…. You will know what to do whereas now, you learn as you go along.’ (P6, female, 5 months in the position)

‘That is what is lacking; training for the post of ward charge.’ (P7, female, 12 months in the position)

‘It is important that the public sector should organise seminars on management; but if you count on the government, you are wasting your time, they do not care. They do not consider many issues. It is not organising seminars on managements that they will consider.’ (P8, male, 4 months in the position)

#### Making sacrifices

Facing difficulties was not the only issue involved in the unit’s management. The transcripts showed that the unit managers reported making sacrifices to cope with their professional responsibilities. For the male unit managers, it was about foregoing other income generating activities.

‘It stops me from having any other activity because I am in the hospital every day; sometimes, even on Saturday, because of my conscientiousness.’ (P8, male, 4 months in the position)

For some female ward charges, it was about having to make additional arrangements for their children to be taken care of as they have to stay longer than before at the hospital to make sure everything is done:

‘We work every day and we do not have much time at home. This means that you have to go the extra mile for someone to take care of your children because you cannot work here and be at home at the same time.’ (P10, female, 5 months in the position)

The participants also mentioned that sacrifices made for the good functioning of the service like buying supplies with their own money:

‘Detergents, gloves, towels; I am the one buying them.’ (P5, female, 5 months in the position)

‘I buy sponges to scour with my money.’ (P2, female, 48 months in the position)

#### ‘It is not worth it’

After facing difficulties and making sacrifices, the participants expected some reward. They agreed that the financial motivation for ward charges in a district hospital was not enough compared to what is required from them. Some expressed their dissatisfaction:

‘What doesn’t make me happy is the salary; because, when I consider all I do compared to what I receive as a ward charge, it is not worth the effort.’ (P5, female, 5 months in the position)

‘I cannot hide it from you; since I was appointed as a ward charge, my salary has not changed at all. What is the use of all this? You work, they ask you to do things, but what should be given to you is not given to you.’ (P6, female, 5 months in the position)

However, other participants admitted that it is not all about money

‘Money should not be more important than your work. A ward charge should consider his job as a form of priesthood, a task done for God. A ward charge who puts money first cannot do his job properly.’ (P7, female, 12 months in the position)

‘It is true that if we were given all the money of the earth, we won’t be satisfied; but the work we do is not small.’ (P3, female, 36 months in the position)

### Assistance from others

The participants had difficulties in their work. The strategies they used to overcome them were to ask for assistance:

‘To improve on the work of a ward charge, we need assistance because it is a job you do relentlessly.’ (P2, female, 48 months in the position)

The assistance they required could be from others, either from the organisation or from their family members or from God. Support from the head nurse was mentioned:

‘Here we hold the unit manager’s meeting with the head nurse to talk about problems we face in our units, among staff; we discuss, and anyone can make a suggestion; it helps.’ (P2, female, 48 months in the position)

‘We ask for the head nurse’s intervention when we can’t deal with issues at our level.’ (P4, male, 4 months in the position)

The participants of this study looked for assistance from their family.

‘I have a fiancé and a little girl. Often when I will be late at home, I am obliged to call so that someone helps her do her homework.’ (P5, female, 5 months in the position)

‘My responsibility remains a sacrifice for my family because this position is time consuming, but my children and their father understand; they give me their full support.’ (P7, female, 12 months in the position)

They expressed it by being grateful for the help they received from their family.

‘Actually, I have a 4-month [*old*] baby; I have somebody at home; my children go to school so my husband helps me a lot; we work together.’ (P9, female, 12 months in the position)

## Discussion

The results revealed that unit managers described their role as difficult. Likewise, male unit managers in Sweden described their role as challenging but driven (Hagerman et al. 2015). The participants of the study reported that they had difficulty leading their staff. The main reasons were the lack of conscientiousness of the staff in relation to their duties, and the illegal sale of drugs. Similarly, Brazilian managers found it difficult to lead their staff because of the lack of commitment of the team members (Amestoy et al. 2014), and the same reason was advanced by South African unit managers in 2014 (Matlakala et al. 2014). In fact, leading in nursing today is a challenging but rewarding opportunity (Bisognano 2016). Unit managers should be at ease and happy to lead; however, if they find it difficult, it could reveal a problem of authority or power. Feelings of powerlessness were also reported by Finnish unit managers (Saarnio, Suhonen & Isola 2016), Swedish unit managers (Hagerman et al. 2015), Australian unit managers (Paliadelis 2013) and Norwegian unit managers (Bentzen, Harsvik & Brinchmann 2013).

The Cameroonian unit managers also felt powerless by admitting that they cannot spank the staff, though physical violence was not the only way of managing conflicts and issues with staff. In Cameroon, corporal punishment of children is still a means to establish authority, and unlike the Australian unit managers who viewed themselves as managers (Ericsson & Augustinsson 2015), the participants of this study viewed themselves as mothers and the staff as children. This could be the reason why spanking was mentioned. Spanking could be a strategy adopted by the participants of this study, while dialogue was adopted to deal with staff-related issues by Armstrong et al. (2015). Dialogue is central to communication, which is part of the directing process in the PODC (Plan, Organise, Direct, Control) framework of unit management (Grohar-Murray, Dicroce & Langan 2016). Also, effective communication with staff is central to effective transmission of information in the unit (Marx 2014). Therefore, specific education in management would help prepare the unit manager to handle the legal, fiscal, planning, directing and controlling functions (Cipriano 2011).

Concerning the illegal or parallel sale of drugs, it is an existing phenomenon in public healthcare facilities in Cameroon (Fusingwa, Payne & Nchang 2013; Zamo-Akono et al. 2013). It ruins the organisation as the drugs are no longer bought at the pharmacy, thus there is less profit. Yet, healthcare organisations want to make a profit like any other organisation. When they consider the inadequate salary of the staff nurses, they sympathise as they feel like the illegal sale of drugs helps the staff to make ends meet.

Unsatisfactory salary was another challenge mentioned. Some participants of this study talked about a pay increase, and that their salary should reflect their workload. Contrarily, in a study by Saarnio et al. (2016), participants admitted that payment increases alone do not motivate people. They argued that more opportunities to create a feeling that their work has meaning have more value than money. The monthly wage paid to a nurse in 2011 was 347–629 US$^1^ in the public sector (Ngah et al. 2013). Not much has changed in terms of salary in Cameroon; yet, a good salary was found to be a good predictor of the unit managers’ intent to stay at the workplace (Van Bogaert et al. 2014). The issue of salary highlights the notion of job satisfaction, which is crucial for the retention of unit managers.

Participants sought assistance and support form co-workers and families and made use of religious coping. Family support was shown to be facilitating resilience among Korean unit managers (Kim & Windsor 2015). Seeking assistance and support from their superiors was reported by unit managers in New Zealand (Gaskin et al. 2012) and in Sweden (Hagerman et al. 2015). Moreover, support was found to be a predictor of a stress-free work environment for unit managers (Van Bogaert et al. 2014). Support is about receiving assistance, collegiality and guidance by various groups within the healthcare organisation (Tuckett et al. 2015). Head managers should be supportive to unit managers to achieve the organisational goals. The organisation should put in place appropriate staffing and other resources for the efficient functioning of the unit (Wong & Laschinger 2015).

The participants of this study were not trained as managers, which could explain why they faced some difficulties. Undoubtedly, unit managers need managerial education and training (Saarnio et al. 2016) as a management position requires a number of leadership skills (Roche et al. 2015).

Furthermore, well-trained and effective unit managers have a positive impact on staff nurses’ working experience and retention (Roche et al. 2015; Townsend et al. 2015). The lack of training in leadership and management can lead to difficulties related to managing the budget (Townsend et al. 2015) or to staffing (Matlakala & Botha 2016). With the current nursing shortages, unit managers who are able to retain staff nurses are in great need. That is why, as the participants suggested, unit managers should be trained in leadership and management prior to appointment. When unit managers are ill-prepared for their tasks, it is likely that they will experience burnout and are less likely to recommend nursing management as a career (Warshawsky & Havens 2014). As recommended by Armstrong et al. (2015), continuing professional development programmes should be developed for unit managers to enhance their management skills and abilities. This will strengthen their roles (Pegram et al. 2014) for better patient, staff and organisational outcomes.

## Recommendations

Because ineffective unit managers harm the staff, patients and the organisation, healthcare organisations should be committed to dedicating sufficient fiscal and human resources to ensuring adequate training and support to unit managers. They should provide for funding and flexible scheduling for unit managers to attend education programmes that will promote the development of required skills, such as conflict management skills. They should also allow the unit managers to have better access to all available resources in the hospital and to provide the resources on time. In addition, the organisations should increase the financial motivation of unit managers because a worker is worthy of his wages. Opportunities should be created for unit managers to express their emotional burdens and for the provision of emotional assistance. Finally, the government should address the issue of illegal drug sales, which is an ethical–legal dilemma.

## Conclusions

Unit management is challenging and strategies can be put in place to overcome the difficulties. The training of unit managers in leadership and management is necessary as a lack of training can narrow the span of control of managers, especially when they do not know that they should be involved in budgeting and in staffing. The full support of head nurses will strengthen the work of the unit manager. Material and supplies should be available for the efficient functioning of the unit. Finally, unit managers should be transformational leaders who can foster staff job satisfaction and reduce the intention of staff members to leave (Munyewende & Rispel 2014).

## Footnotes

1 Based on an exchange rate of US$1 = 500CFA.
